# Elongation Factor 1β′ Gene from *Spodoptera exigua*: Characterization and Function Identification through RNA Interference

**DOI:** 10.3390/ijms13078126

**Published:** 2012-06-29

**Authors:** Li-Na Zhao, Zi Qin, Ping Wei, Hong-Shuang Guo, Xiang-Li Dang, Shi-Gui Wang, Bin Tang

**Affiliations:** 1 Hangzhou Key Laboratory of Animal Adaptation and Evolution, College of Life and Environmental Sciences, Hangzhou Normal University, Hangzhou 310036, China; E-Mails: heping895784491@yeah.net (L.-N.Z.); qinzi110@163.com (Z.Q.); njnnwp@yahoo.cn (P.W.); wybghs@126.com (H.-S.G.); sgwang@mail.hz.zj.cn (S.-G.W.); 2 Zhejiang Institute of Subtropical Crops, Wenzhou, Zhejiang 325005, China; E-Mail: xianglidang@yahoo.com.cn

**Keywords:** elongation factor, cloning, expression pattern, RNAi, *Spodoptera exigua*

## Abstract

Elongation factor (EF) is a key regulation factor for translation in many organisms, including plants, bacteria, fungi, animals and insects. To investigate the nature and function of elongation factor 1β′ from *Spodoptera exigua* (SeEF-1β′), its cDNA was cloned. This contained an open reading frame of 672 nucleotides encoding a protein of 223 amino acids with a predicted molecular weight of 24.04 kDa and pI of 4.53. Northern blotting revealed that *SeEF-1β′* mRNA is expressed in brain, epidermis, fat body, midgut, Malpighian tubules, ovary and tracheae. RT-PCR revealed that *SeEF-1β′* mRNA is expressed at different levels in fat body and whole body during different developmental stages. In RNAi experiments, the survival rate of insects injected with *SeEF-1β′* dsRNA was 58.7% at 36 h after injection, which was significantly lower than three control groups. Other elongation factors and transcription factors were also influenced when EF-1β′ was suppressed. The results demonstrate that *SeEF-1β′* is a key gene in transcription in *S. exigua*.

## 1. Introduction

Initiation, elongation and termination are the three main steps in translation. Elongation factor is a highly conserved protein that plays a role in peptide elongation during translation [1–4] and is required for protein biosynthesis, with effects such as regulation of protein biosynthesis and acceleration of apoptosis [5].

In all living organisms, the translational machinery requires a minimum set of at least 20 aminoacyl-tRNAs (aa-tRNAs), one for each of the standard amino acids (aa) found in proteins. Most aa-tRNAs are formed by direct attachment of aa to homologous tRNA by the cognate aa-tRNA synthetase (aaRS) [6,7]. Although initiation is the most highly regulated step, recent work highlights the crucial regulation of translation elongation in controlling mRNA levels. Translation requires the specific attachment of amino acids to tRNAs by aaRS and subsequent delivery of aa-tRNAs to the ribosome by elongation factor. Regulation of translation elongation controls not only the continuous and ubiquitous expression of immediate early genes, but also the expression of a large number of gene transcripts, which may be arrested in certain rapidly reversible conditions such as starvation [8–11]. Furthermore, translation elongation is directly linked to transcript maturation (capping, splicing, polyadenylation) [8–10].

Elongation factor was first identified and isolated from *Escherichia coli* in the early 1960s. Protein biosynthesis in prokaryotes requires three types of elongation factors, EFTu, EFTs and EFG, with a molecular weight of 47, 36 and 83 kDa, respectively. Elongation factors Tu and G are the bacterial counterparts of universal transcription factors and are members of the G protein superfamily [12–14], but elongation factors 1 (eEF1), eEF2 and eEF3 are essential for peptide chain elongation during translation in eukaryotes [2,15]. The role of eEF-1A (eEF-1α) in protein biosynthesis has been extensively studied. EF-lA plays an essential role in protein biosynthesis in eukaryotic cells [1]. The protein transfers charged tRNAs into the unoccupied acceptor site of the ribosome in a step that requires GTP. Recent data for EF-Tu, the analogous elongation factor in prokaryotic cells and eukaryotic organelles, suggest that this protein is partly responsible for proofreading the interaction of codon and anticodon, suggesting that it plays a direct role in the fidelity of translation [15,16].

Elongation factor genes have been extensively studied in plants, bacteria and fungi, but not in insects. In insects, all four types of elongation factors have been cloned or deduced from genomic sequences (according to GenBank). In the present study, we cloned cDNA of the elongation factor 1β′ gene from *Spodoptera exigua* (*SeEF-1β′*, accession no. EU258621). The tissue distribution and expression patterns of this gene were investigated. Moreover, RNA interference (RNAi) was used to study the function of the gene.

## 2. Results and Discussion

### 2.1. Sequence Analysis of *SeEF-1β′* cDNA

**Figure 1:**
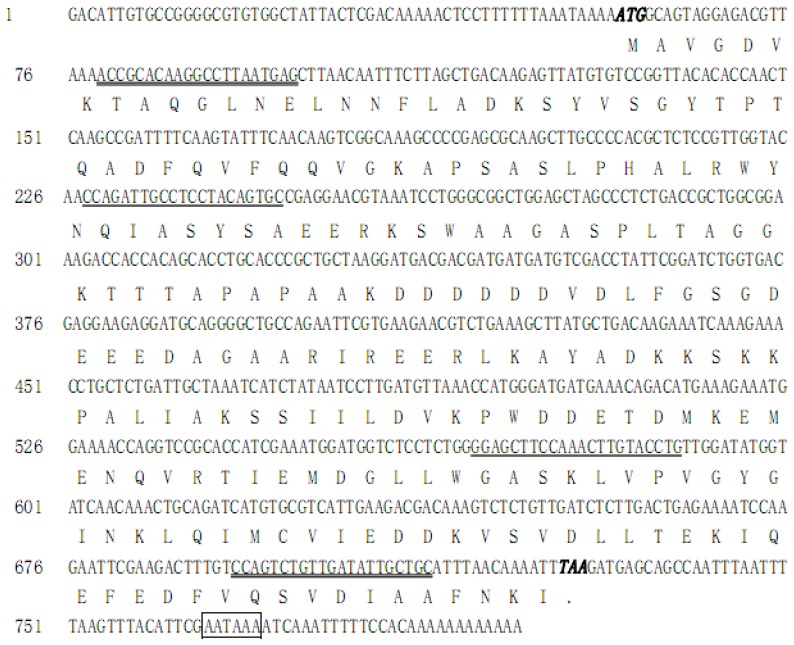
Nucleotide and amino acid sequences of *EF-1β′* from the beet armyworm *S. exigua*. The nucleotide sequence reported in this paper has been submitted to GenBank under accession no. EU258621. Italic and overstriking nucleotides are start and stop codons, respectively. The primers for *dsSeEF-1β′* RNA synthesis and detection are marked by an underline and double underline, respectively. The termination signal AATAAA is boxed.

**Figure 2:**
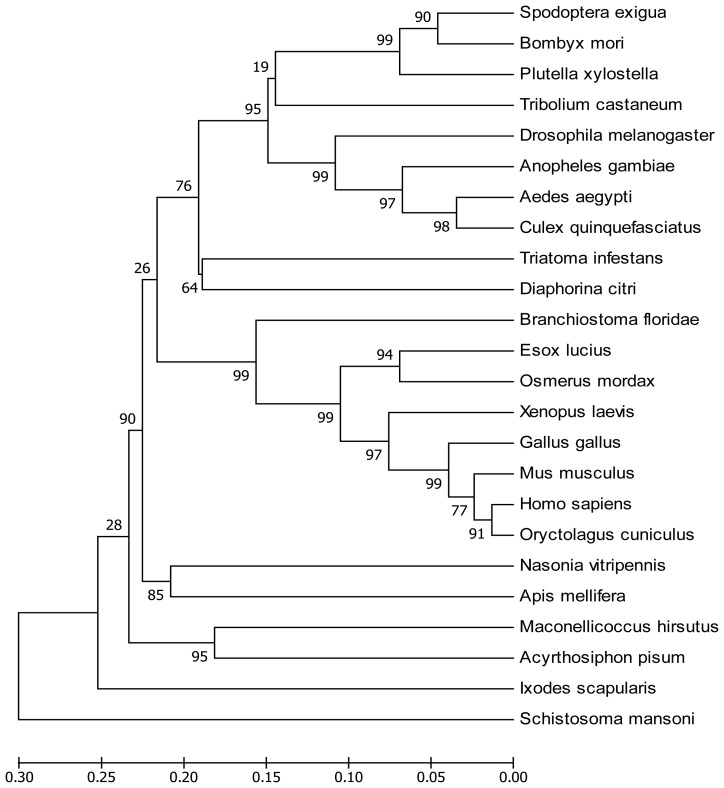
Phylogenetic analysis of SeEF-1β′ and EF-1β′s from other animal species. The phylogenetic tree was constructed based on the amino acid sequences of known EFs. Full length amino acid sequences were aligned with the Mega 3.1 program to generate the phylogenetic tree. A bootstrap analysis was carried out, and the robustness of each cluster was verified with 1000 replicates. Values at the cluster branches indicate the results of the bootstrap analysis. The scale on the x-axis represents the estimated branch lengths. *EF-1β′s* were from *A. pisum* (NM_001162346), *A. aegypti* (AY552052), *A. gambiae* (XM_558148), *A. mellifera* (XM_625024), *B. mori* (NM_001044091), *B. floridae* (XP_002227182), *C. quinquefasciatus* (XM_001847313), *D. citri* (DQ673433), *D. melanogaster* (NM_080069), *E. lucius* (BT079178), *G. gallus* (AJ721003), *H. sapiens* (CH236950), *I. scapularis* (DQ066216), *M. hirsutus* (EF070471), *M. musculus* (AK012756), *N. vitripennis* (XM_001599881), *O. cuniculus* (NM_001082399), *O. mordax* (BT074513), *P. xylostella* (AB180443), *S. mansoni* (FN357357), *S. exigua* (EU258621), *T. infestans* (EF639083), *T. castaneum* (XM_968676)and *X. laevis* (NM_001090665) (all sequences from GeneBank).

*SeEF-1β′* cDNA (accession no. EU258621) was obtained by PCR and RACE. *SeEF-1β′* cDNA has an open reading frame of 672 nucleotides (Figure 1), which encodes a protein of 223 amino acids with a predicted mass of approximately 24.04 kDa and a pI of 4.53. SeEF-1β′, which shows 52–89% identity to other known EF-1β forms and insect EF-1β, can be clearly distinguished from those of animals (Figure 2).

**Figure 3:**
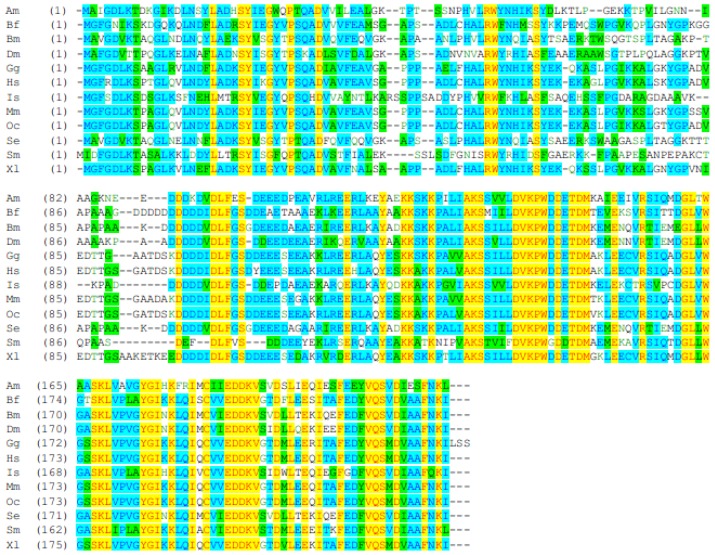
Alignment of EF-1β′ proteins from insects and other animals. EF-1β′s were from *A. mellifera* (Am), *B. mori* (Bm), *B. floridae* (Bf), *D. citri* (Dc), *D. melanogaster* (Dm), *G. gallus* (Gg), *H. sapiens* (Hs), *I. scapularis* (Is), *M. musculus* (Mm), *O. cuniculus* (Oc), *S. mansoni* (Sm), *S. exigua* (Se), and *X. laevis* (Xl). GenBank accession numbers (DNA) are as in Figure 2. Highly conserved regions are shown in yellow, green and sky-blue.

The deduced amino acid sequence of SeEF-1β′ was aligned with sequences from other species. SeEF-1β′ is most similar to the EF-1β′ of *Bombyx mori* (90% identity, Figure 2). It is also similar to EF-1β′ of *Plutella xylostella* (83%), *Anopheles gambiae* (76%), *Aedes aegypti* (73%), *Drosophila melanogaster* (73%), *Culex quinquefasciatus* (72%), *Tribolium castaneum* (71%), *Diaphorina citri* (66%), *Triatoma infestans* (64%), *Maconellicoccus hirsutus* (64%), *Xenopus laevis* (62%), *Nasonia vitripennis* (62%), *Acyrthosiphon pisum* (62%), *Branchiostoma floridae* (62%), *Apis mellifera* (60%), *Esox lucius* (60%), *Osmerus mordax* (60%), *Mus musculus* (59%), *Gallus gallus* (59%), *Oryctolagus cuniculus* (58%), *Homo sapiens*(57%), *Ixodes scapularis* (55%) and *Schistosoma mansoni* (52%). Multiple sequence alignment of EF-1β′ proteins showed a high degree of conservation, particularly in the middle of the putative catalytic domain (Figure 3).

Alignment of EF-1β of insects and other animals revealed two conserved motifs, DVKPWD/GDE/DTDM and EDDKV, which may be signature sequences (Figure 3). However, insect EF-1β contains four conserved motifs: DVDLF, IAKSS, D/EVKPWDDETD/NM and VQSVDI.

### 2.2. Tissue Distribution and Developmental Expression of *SeEF-1β′*

**Figure 4:**
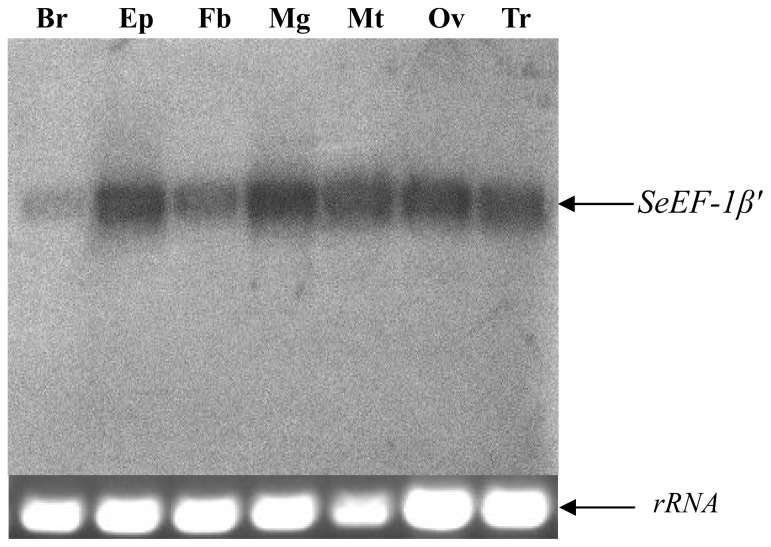
Northern blot analyses of the *SeEF-1β′* transcript in different tissues of fifth instar *S. exigua* larvae. Total RNA was extracted from various tissues: Brain (Br), Epidermis (Ep), Fat body (Fb), Midgut (Mg), Malpighian tubules (Mt), Ovary (Ov) and Tracheae (Tr). An *SeEF-1β′*-specific probe was radiolabeled with (α-^32^P)-dCTP. Following hybridization and detection by autoradiography using the *SeEF-1β′* probe, the membrane was stripped by boiling in 0.1% SDS. The rRNA was used as a reference.

**Figure 5:**
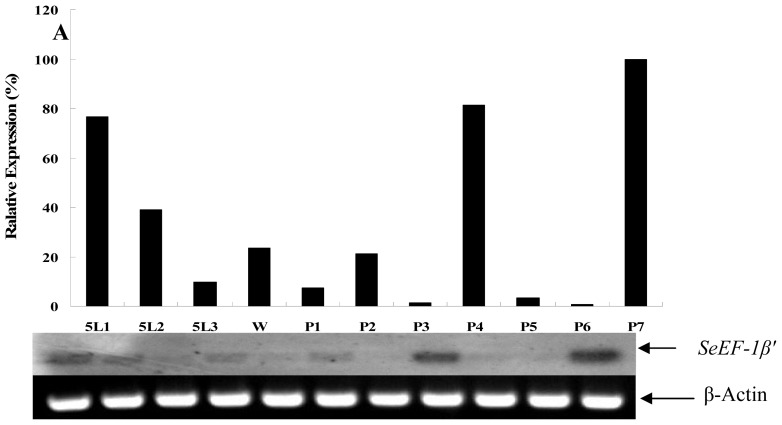

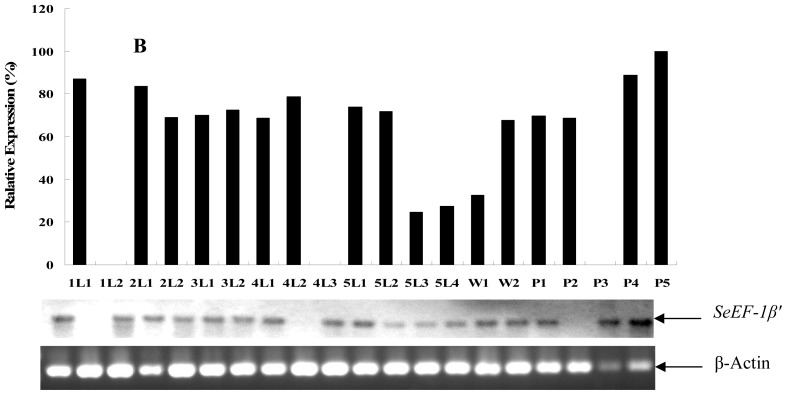
Developmental expression of *S. exigua EF-1β′* mRNA in the fat body (**A**) and whole body (**B**). [α-^32^P] dCTP labeled *SeEF-1β′* cDNA, which was amplified using specific primers EF-FP and EF-RP, was used as a probe. β-actin was labeled with [α-^32^P] dCTP as a control. (**A**) RNA was extracted from the fat body every 24 h from fifth instar larvae (5L), wandering (pre-pupae) larvae (W) and pupae (P) 5L1-5L3 means the first to third day of the fifth instar larvae. W means pre-pupae larvae; P1-P7 means the first to seventh day of papae; (**B**) RNA was extracted from the whole body from first instar larvae to the fifth day of pupae (P). 1L1-5L4 means the first day of the first instar larvae to fourth day of the fifth instar larvae; W1 and W2 means the first and second day of pre-pupae larvae; P1-P5 means the first to fifth day of pupae. β-Actin was used as a loading control.

Northern blotting revealed that *EF* was expressed in brain, epidermis, fat body, midgut, Malpighian tubules, ovary and tracheae (Figure 4). RT-PCR were carried out to analyze the expression patterns of *SeEF-1β′* in fat body (from fifth instar larvae to pupae) and whole body (from first star larvae to pupae) during *S. exigua* development. The results showed that *SeEF-1β′* mRNA was expressed in fat body and whole body at different levels from fifth instar larvae to pupae. *SeEF-1β′* transcripts were highly expressed in fat body on day 1 of fifth instar larvae, as well as on days 4 and 7 of the pupal stage. Transcripts were present at lower levels in fat body on day 3 of fifth instar larvae and days 1, 3, 5 and 6 of the pupal stage (Figure 5A). *SeEF-1β′* transcripts were highly expressed in whole body in all developmental stages, with lower levels detected on days 3 and 4 of fifth instar larvae and pre-pupa, and no expression on day 2 of first instar larvae, day 3 of fourth instar larvae and day 3 of the pupal stage (Figure 5B). The results indicated that *SeEF-1β′* mRNA was constitutively expressed at different levels during developmental stages.

### 2.3. The Survival Rate and Expression of *SeEF-1β′* after dsRNA Injection

**Figure 6:**
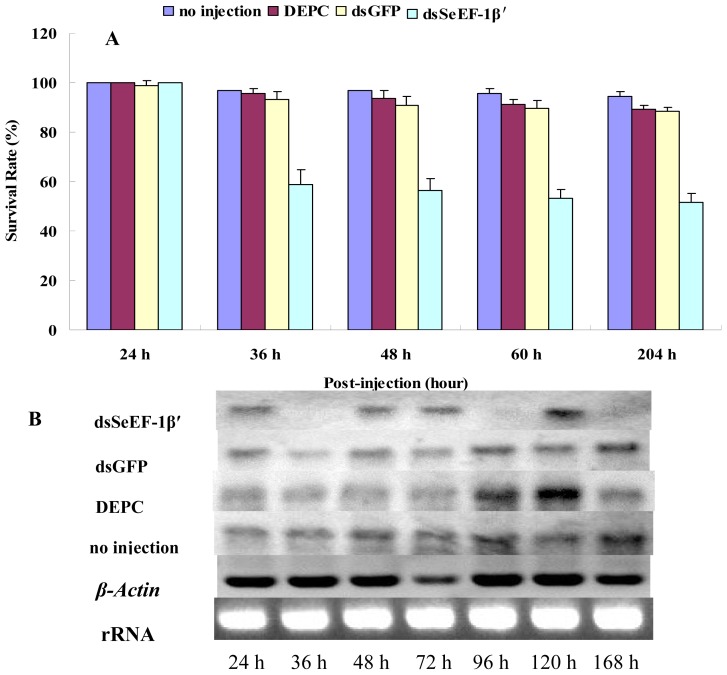
*S. exigua* survival rates and RT-PCR analysis of *SeEF-1β′* transcripts after injection of dsRNA. (**A**) Survival rates of insects at different times after injection of *dsSeEF-1β′*, *dsGFP*, DEPC water alone and no injection. Survival rates of insects based on key developmental stages for time intervals of 24 h, 36 h, 48 h (pre-pupae stage), 60 h (pupation stage) and 204 h (eclosion stage) post-injection. Percentage values were arcsine square root transformed prior to analyses to correct for the non-normal distribution of percentage values. Different letters in the same injection stage indicate significant difference of the survival rates (*p*, 0.05, Duncan’s test). No significant difference was found by ANOVA (*p*, 0.05). All error bars represent standard deviation (*n* = 3); (**B**) Three lively insects were obtained at 24 h, 36 h, 48 h, 72 h, 96 h, 120 h and 168 h after injection at random. Total RNA was extracted and *SeEF-1β′* transcripts were detected using RT-PCR. The post-injection time for 24 h, 36 h, 48 h, 72 h, 96 h, 120 h and 168 h are marked. The rRNA and housekeeping gene of β-actin were used as references.

*DsSeEF-1β′* was injected on day 2 of the 5th instar stage (just 24 h after pupation) advantageous for injection. After 36 hours, the injection caused a lethality rate of 41.3%, as exhibited by body softness followed by death. By contrast, few larvae died or exhibited abnormal phenotypes in dsGFP (Green Fluorescent Protein), DEPC (diethypyrocarbonate) water and control groups (Figure 6A).

The survival rate of insects injected with *dsSeEF-1β′* was 58.70%, 56.36%, 53.14% and 51.64% at 36 h, 48 h, 60 h and 204 h post-injection, respectively, which is significantly lower than the rate for the three control groups (93.18%, 90.88%, 89.73% and 88.54% for dsGFP injection, 95.73%, 93.58%, 91.39% and 89.27% for DEPC water injection and 96.66%, 96.66%, 95.55% and 94.37% for no injection) (Figure 6A). RT-PCR revealed that *SeEF-1β′* expression was lower in the treated group than in the three control groups at 36 h and 96 h after injection (Figure 6B).

### 2.4. The Expression of EF-1α, EF-2 and Fork Head mRNA after Injection of dsSeEF-1β′

**Figure 7:**
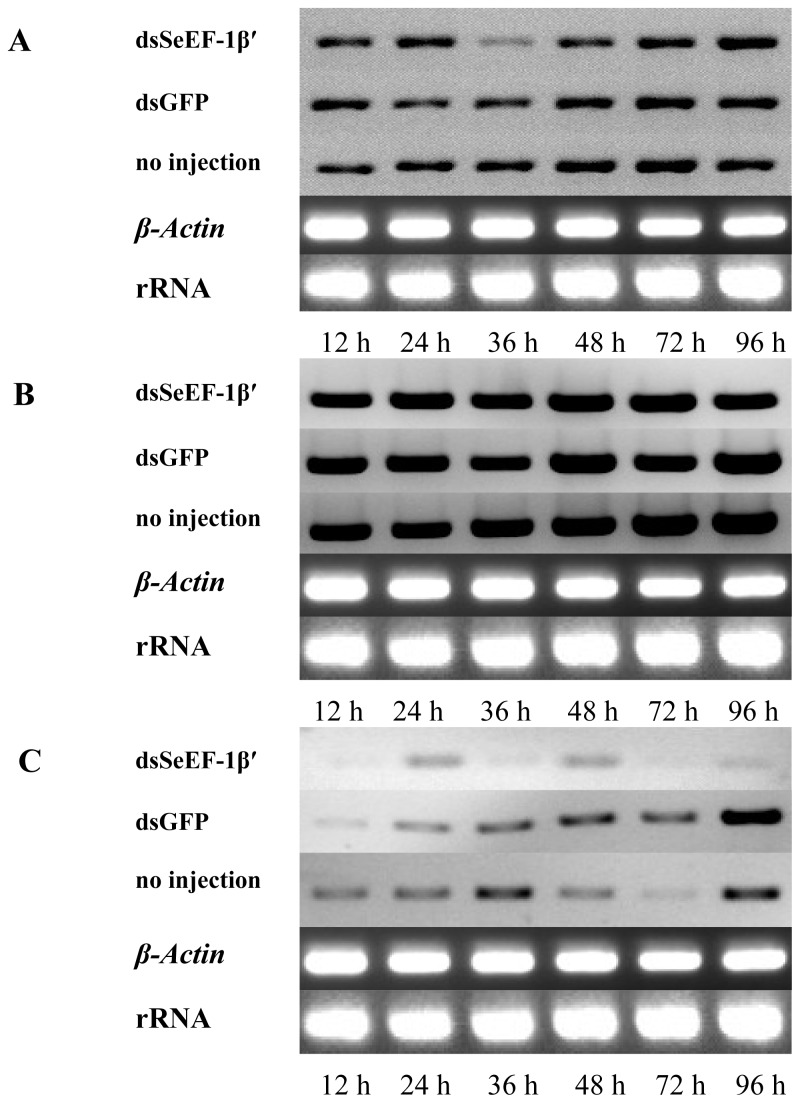
RT-PCR analysis of *SeEF-1α*, *SeEF-2* and *SeFH* mRNA expression after injection of *ds SeEF-1β′*. Three lively insects were obtained at 12 h, 24 h, 36 h, 48 h, 72 h and 96 h after injection at rando. Total RNA was extracted and *SeEF-1α*, *SeEF-2* and *SeFH* transcripts were detected using RT-PCR. The rRNA and housekeeping gene of β-actin were used as references. (**A**) *SeEF-1α* gene’s expression after injected dsSeEF-1β′, dsGFP and no injection; (**B**) *SeEF-2* gene’s expression after injected dsSeEF-1β′, dsGFP and no injection; (**C**) *SeFH* gene’s expression after injected dsSeEF-1β′, dsGFP and no injection.

Day-2 fifth instar larvae were used as experimental insects for injection of *dsSeEF-1β′* treatments. RT-PCR, *SeEF-2*, *SeFH* injected with *dsSeEF-1β′* groups displayed lower expression after 36 h compared with *dsGFP* and no injection groups (Figure 7A–C).

EFs facilitate the ribosome to synthetize protein. Previous studies identified two universally conserved transcription factors EFs, EF-Tu in bacteria (known as eEF1A in eukaryotes) and EF-G (eEF2), which deliver aa-tRNAs to the ribosome and promote ribosomal translocation, respectively [2,3,15]. The eEF-1 family consists of four different subunits, EF-1α (51 kDa), EF-1β′ (26 kDa), EF-1β (33 kDa) and EF-1γ (49 kDa) [17,18]. The EF-1 complex catalyzes the exchange of EF-1α-bound GDP for exogenous GTP. Both eEF1-β′ and eEF-1β possess guanine nucleotide exchange activity [19,20]. eEF-1γ acts in tandem with eEF1-β′ to facilitate exchange of eEF-1α-bound GDP for GTP [21]. eEF-1α has been extensively investigated in bacteria, plants, insects and animals [7,22–24]. It is not only required during the elongation phase of transcription, but also functions as a cross-linker for F-actin [22–25]. It is used as a molecular marker of species evolution and it has been reported that eEF-1α overproduction suppresses the peroxisome-deficient phenotype of a *Hansenula polymorpha* pex3-1 mutant [24]. In yeast, termination of translation is controlled by two interacting polypeptide chain release factors, eRF1 and eRF3, and eEF1β can modulate the functions of eRF1 and eRF3 and the efficiency of translation termination [26].

In insects, *EF-1β′* cDNA was first cloned from *B. mori* silk gland in 1992 [27]. *EF-1α* cDNA was cloned and was found to be very similar to *E. coil EF-Tu* [28]. Silk gland *EF-1β* from *B. mori* cDNA has been cloned and contains an open reading frame encoding a polypeptide of 423 amino acids and shares 67.3% amino acid identity with *EF-1β* from *Artemia salina*. Kamiie and colleagues demonstrated that the EF-1β *N*-terminal domain is 29.3% identical to maize glutathione *S*-transferase and bound to glutathione [29]. So far, *EF-1β′* cDNA has been cloned or deduced from genomic sequences for many insects; however, its tissue distribution and expression patterns in fat body and other tissues are still unknown. It is well known that almost all of genes were expressed in the fat body, which is the center of material metabolism. In the present study, we found that *EF-1β′* is expressed in almost all *S. exigua* tissues (Figure 4) and at different levels in the fat body (Figure 5A) and whole body (Figure 5B) in different developmental stages.

It is reported that *BmEF-1β′* can bind to glutathione Sepharose, which suggested that it’s *N*-terminal domain has the capacity to bind to glutathione [27,29]. We cloned and characterized cDNA for *EF-1β′* from *S. exigua.* Analysis of EF-1β′ protein sequences revealed two conserved motifs, DVKPWD/GDE/DTDM and EDDKV, which may be pivotal sites in EF-1β′ in animals (Figure 3). Four conserved motifs were identified in insect EF-1β′ forms: DVDLF, IAKSS, D/EVKPWDDETD/NM and VQSVDI. The results indicate that DVDLF, IAKSS and VQSVDI sequences may have important functions in insect transcription.

Protein transcription is a key process for protein synthesis [5,9,25]. RNAi is a good approach for investigating gene function. Wang and colleagues revealed that transcription elongation can control cell fate in Drosophila embryos [30]. In the present study we investigated whether *S. exigua* could survive and develop after *EF-1β′* was knocked down. Approximately 41.3% insects exhibited body softness and subsequently died at 36 h after *dsSeEF-1β′* injection (Figure 6A), which may be explained by the failed expression of many genes. RT-PCR revealed that *SeEF-1β′* mRNA levels were much lower at 36 h after *dsSeEF-1β′* treatment compared to the three control groups. *SeEF-1β′* mRNA injected with *dsSeEF-1β′* was also lower at 96 h and 168 h compared to the control, but insect growth was normal.

Fork head (SeFH) is a general translation factor, and SeEF-1α and SeEF-2 are elongation factors. The expression of these three factors was also detected by RT-PCR. The results showed *SeEF-1α* and *SeEF-2* genes’ expression were similar among *dsSeEF-1β′* treatment, *dsGFP* and no injection groups, but *SeFH* gene’s expression was lower after *dsSeEF-1β′* treatment compared with the other two groups. At the same time, *SeEF-1β′* displays about 30% similarity with *SeEF-1α* and *SeEF-2* from sequence alignments. Thus, the expression of *SeEF-1α* and *SeEF-2* cannot be silenced by the *dsSeEF-1β′* treatment. These results also suggest that suppression of *EF-1β′* can also affect the regulation of the other elongation factors or transcription factors.

## 3. Experimental Section

### 3.1. Insect Cultures

*S. exigua* larvae were reared at 26 °C with an L14:D10 photoperiod using an artificial diet [31–33]. The developmental stages were synchronized at each molt by collecting new larvae or pupae. The brain, midgut, fat body, epidermis, Malpighian tubules, ovary and tracheae from fifth instar larvae to pupae and whole body from all stages were dissected in a 0.75% NaCl solution and stored at −80 °C until further use.

### 3.2. RNA Isolation, cDNA Synthesis and PCR

Total RNA was isolated from the fat body of *S. exigua* pupae using the acid guanidinium thiocyanate-phenol-chloroform method [34].

Three degenerated primers, EF-F1 (5′-GGH GAC GTB AAV ACC GC-3′, sense), EF-F2 (5′-AAG AAR TCN AAG AAA CC-3′, sense), and EF-R (5′-GCD GCA ATG TCV ACA GA-3′, anti-sense), were designed based on the conserved amino acid sequences of known EFs. The first PCR reaction was performed with primers EF-F1 and EF-R using the following conditions: three cycles of 40 s at 94 °C, 40 s at 45 °C and 60 s at 72 °C followed by 30 cycles of 40 s at 94 °C, 40 s at 48 °C and 60 s at 72 °C. A second PCR was carried out using the nested primers EF-F2 and EF-R under the same conditions as for the first PCR [33,35–37]. The expected band was purified using a DNA gel extraction kit (Takara, Japan) and cloned into the pMD18-T vector (Takara) and sequenced by the dideoxynucleotide method (Takara).

### 3.3. Rapid Amplification of cDNA Ends (RACE)

For 5′- and 3′-RACE, cDNAs were synthesized according to the manufacturer’s protocol (SMART™ kit, Clontech). Specific primers EF-5R1 (5′-CCA TGG TTT AAC ATC AAG G-3′, anti-sense) and EF-5R2 (5′-GAT TTA GCA ATC AGA GCA GG-3′, anti-sense) for 5′-RACE and EF-3F1 (5′-CTG CAG ATC ATG TGC GTC-3′, sense) and EF-3F2 (5′-GTC TCT GTT GAT CTC TTG-3′, sense) for 3′-RACE were synthesized based on the cDNA sequence of the PCR fragment.

### 3.4. cDNA and Protein Sequence Analyses

The *SeEF-1β′* cDNA sequence was compared with other *EF* sequences deposited in GenBank using the BLAST-N and BLAST-X tools on the National Center for Biotechnology Information (NCBI) website. The amino acid sequence of *SeEF-1β′* was deduced from the corresponding cDNA sequence using the transcription tool on the ExPASy Proteomics website [38] A phylogenetic tree was constructed using MEGA 5.05 software based on the amino acid sequences of known EFs. A bootstrap analysis was carried out and the robustness of each cluster was verified using 1000 replicates. Other protein sequence analysis tools on the ExPASy Proteomics website [38] were used to determine the molecular weight, pI and *N*-glycosylation sites. Multiple sequence alignment of insect EFs was performed using the tool at the multiple sequence alignment website [39]

### 3.5. Northern Blot

Samples of 25 μg of total RNA isolated from midgut, brain, Malpighian tubules, epidermis, fat body, tracheae and ovary of fifth instar larvae were separated on a formaldehyde agarose gel containing ethidium bromide. The RNA was subsequently blotted onto a Hybond-N+ membrane (Amersham). A cDNA fragment of 635 bp with the EF-FP (5′-ACC GCA CAA GGC CTT AAT GAG-3′, sense) and EF-RP (5′-GCA GCA ATA TCA ACA GAC TGG-3′, anti-sense) primers was labeled with [α-^32^P]-dCTP using a random primer DNA labeling kit (Takara, Japan) and then used as the hybridization probe. Membranes were pre-hybridized at 42 °C for 4 h, followed by addition of the α-^32^P-labeled *SeEF-1β′* probe at 42 °C for 18 h in 5× SSPE containing 50% formamide, 5× Denhardt’s solution, 0.1% SDS and 100 mg/mL salmon sperm DNA. After hybridization, the membrane was washed with 0.2× SSPE at 45 °C and exposed to X-ray film at −70°C for 24 h [32,33,40].

### 3.6. Determination of Developmental Expression of SeEF-1β′ by RT-PCR Analysis

The fat body of fifth instar larvae, pre-pupae and pupae and the whole body of first, second, third, fourth and fifth instar larvae, pre-pupae and pupae were dissected. Total RNA was isolated from the fat body of 11 stages and the whole body of 20 stages and 1 μg of total RNA from each sample was reverse transcribed at 42 °C for 1 h in a final volume of 10 μL containing reaction buffer, 10 mM DTT, 0.5 mM dNTP, 0.5 mg of oligo-dT18, and AMV reverse transcriptase.

RT-PCR reactions were performed with the EF-FP/EF-RP primers and total RNA of the fat body and whole body treated with DNase was used as templates under the following conditions: 30 cycles of 40 s at 94 °C, 40 s at 55 °C and 60 s at 72 °C. Each PCR product (5 μL) was electrophoresed and detected by ethidium bromide staining. The amount of *S. exigua β-actin* per lane was used as a loading control.

### 3.7. Injection of dsSeEF-1β′ into *S. exigua* Larvae

DsRNA corresponding to *SeEF-1β′* (*dsSeEF-1β′*) was prepared using a T7 RiboMAX™ Express RNAi System (Promega, USA) according to a previously established method [41]. Larvae at 24 h after the fifth instar stage were used for injection experiments because larvae in earlier stages of development were too small for satisfactory injection. A sample of 5μg of dsRNA dissolved in 5 μL of DEPC water was injected into the side of the thorax of *S. exigua* larvae using a 10 μL syringe (Hamilton) and the injection point was immediately sealed with wax. Control larvae were injected with 5 μg of dsRNA dissolved in 5 μL of DEPC water corresponding to a *GFP* gene (*dsGFP*), 5 μL DEPC water alone or was not injected. Each group comprised 30 individual larvae, the total RNA from the whole body of groups of five larvae were used in RT-PCR.

### 3.8. Observation of Insect Survival and Data Analysis

Larvae were observed at 12 h intervals after treatment to identify deaths, size differences, slow action and other abnormal changes among the groups. To test for an effect of treatment, ANOVAs were performed using the cumulative percentage of abnormal and dead larvae as the dependent variable and group (no injection, DEPC water injection, *dsGFP* injection, *dsSeEF-1β′* injection) as the independent variable. Post-hoc Duncan’s tests were used to determine differences among groups when treatment effects were detected. These analyses were repeated at 24 h, 36 h and 48 h (pre-pupae stage), 60 h (pupation stage) and 204 h (eclosion stage) post-injection. Percentage values were arcsine square-root-transformed prior to analyses to correct for non-normal distribution.

### 3.9. RT-PCR Analysis of *EF-1β′* Gene Silencing and *EF-1α, EF-2* and Fork Head (*FH*) mRNA Expression

Insects (including larvae, pupae and adults) were observed and sampled at 12, 24, 36, 48, 72, and 96 h after injection. Three lively larvae were removed at random and stored at −80°C for subsequent RNA extraction. Total RNA was extracted from individual larvae using AMV reverse transcriptase. The EF-FP and EF-RP primers were used to amplify cDNAs in the same PCR reactions. Pilot experiments demonstrated that 22–24 cycles were optimal for linear amplification of the PCR products, and this protocol was then used in subsequent experiments. PCR amplification was performed in a 25 μL reaction mixture using the following conditions: 10 min at 94 °C; 22–24 cycles of 1 min at 94 °C, 1 min at 60 °C and 1 min at 72 °C; followed by 10 min at 72 °C. The PCR products were separated on a 2% agarose gel and transferred to a Hybond-N+ nylon membrane. Hybridization, washing and signal detection of the blots were similar to the procedures described previously [42].

The EF1α-FP (5′-CTC TTA CAT CAA GAA GAT CG-3′, sense), EF1α-RP (5′-GGA CTT GGG GTT GTC CTC-3′, anti-sense), EF2-FP (5′-GAC TGT GTC TCA GGT GTG TG-3′, sense), EF2-RP (5′-GGT CGC AGT TCT TGA TAC C-3′, anti-sense), FH-FP (5′-GAC TGC TTC GTG AAA GTG CC-3′, sense) and FH-RP (5′-CGT CGT ACA TCT TCA GGT CTG C-3′, anti-sense) primers were used to amplify cDNAs in the same PCR reactions. Pilot experiments demonstrated that 30 cycles were optimal for linear amplification of the PCR products, and these PCR products were separated and color developed on a 1.5% agarose gel electrophoresis.

## 4. Conclusions

The study demonstrated that *SeEF-1β′* is a housekeeping gene. *SeEF-1β′* is constitutively expressed in all *S. exigua* tissues during developmental stages. *DsSeEF-1β′* can clearly reduce the survival rate by directly influencing the expression of *SeEF-1α*, *SeEF-1β′*and *SeFH*.
